# Constant Levels of Tau Phosphorylation in the Brain of htau Mice

**DOI:** 10.3389/fnmol.2020.00136

**Published:** 2020-08-28

**Authors:** Joerg Neddens, Magdalena Daurer, Tina Loeffler, Saioa Alzola Aldamizetxebarria, Stefanie Flunkert, Birgit Hutter-Paier

**Affiliations:** QPS Austria GmbH, Grambach, Austria

**Keywords:** tauopathies, Alzheimer’s disease, human tau, phosphorylation, mouse model, immunofluorescent labelling, genetic background, progression

## Abstract

Excessive tau phosphorylation is the hallmark of tauopathies. Today’s research thus focusses on the development of drugs targeting this pathological feature. To test new drugs in preclinical studies, animal models are needed that properly mimic this pathological hallmark. The htau mouse is a well-known model expressing human but lacking murine tau, allowing to evaluate the efficacy of tau modifying compounds without interference from murine tau. Htau mice are well-characterized for tau pathology at older age, although it is often not specified on which genetic background analyzed animals were bred. Since it was shown that the genetic background can influence the pathology, we evaluated the phosphorylation status of young and adult htau mice on a C57BL/6J background by analyzing ptau Ser202 and ptau Ser396 levels in the cortex and hippocampus of 3 and 12 month old animals by immunofluorescent labelling. Additionally, we evaluated total tau, ptau Thr231 and ptau Thr181 in the soluble and insoluble brain fraction of 3–15 month old htau mice by immunosorbent assay. Our results show that ptau levels of all analyzed residues and age groups are similar without strong increases over age. These data show that tau is already phosphorylated at the age of 3 months suggesting that phosphorylation starts even earlier. The early start of tau phosphorylation in htau mice enables the use of these mice for efficacy studies already at very young age.

## Introduction

Tauopathies are a group of diseases that are all characterized by neuropathological phosphorylation and aggregation of the microtubule associated protein tau (MAPT). The long list of tauopathies includes, but is not limited to, Alzheimer’s disease (AD), Down’s syndrome, some prion disease variants, progressive supranuclear palsy, amyotrophic lateral sclerosis/parkinsonism-dementia complex of Guam, Pick’s disease, corticobasal degeneration, and frontotemporal dementia with parkinsonism (Lee and Trojanowski, 1999). Current research efforts try to treat these tauopathies using active or passive immunization approaches as well as glycogen synthase kinase 3 or heat shock protein 90 inhibitors (Boutajangout and Wisniewski, 2014; Sigurdsson, 2018) but the success is still limited. Highly reliable *in vivo* models that properly mimic human tau pathology are essential for efficacy studies to be able to translate preclinical results into the clinic. The htau transgenic mouse features expression of all six isoforms of human tau combined with a knockout of murine tau and thus allows testing of humanized compounds without interference of murine tau (Andorfer et al., 2005). These mice therefore belong to the best animal models to test compounds directed against human tau (Andorfer et al., 2005). The original publication describes htau mice to show strong tau phosphorylation at residues Ser202 and Ser396/404 at the age of 16 months but lacking neurofibrillary tangles (NFTs) (Andorfer et al., 2005). Animals further present extensive cell death as analyzed by TUNEL staining, measurement of cortical thickness, ventricle size and number of neuronal cells (Andorfer et al., 2005). Geiszler et al. (2016) presented data that suggest an influence of the genetic background on the neuropathological features of htau mice. Their results argue for a weakened phenotype in htau mice that were backcrossed to a C57BL/6J background (Polydoro et al., 2009), while the original publication of these backcrossed htau mice showed still visual recognition memory and spatial memory deficits in 12 month old animals. Additionally, backcrossed mice showed progressively increasing levels of pSer202 tau and pSer396/404 tau as analyzed by qualitative histological evaluations in 4 and 12 month old htau mice (Polydoro et al., 2009). Although tau phosphorylation (ptau) was already extensively studied in old htau mice, it is not mentioned in these reports on which background analyzed animals were bred (Acker et al., 2013; Forest et al., 2013). We therefore evaluated the cortex and hippocampus of young and old htau mice on a C57BL/6J background for ptau Ser202 and ptau Ser396 by immunofluorescent labeling followed by rater-independent macro-based signal quantification. Additionally, we quantitatively analyzed soluble and insoluble brain levels of total tau, ptau Thr231 and ptau Thr181 in htau mice of four age groups ranging from 3 to 15 months. Our results show phosphorylation of tau at residue Ser202 and Ser396 already at 3 month of age that barely changes in older animals. Furthermore, we demonstrate a minor increase of total tau, ptau Thr231 and ptau Thr181 in the soluble but not insoluble brain fraction over age.

## Materials and Methods

### Animals

Htau mice express human tau derived from a human PAC, H1 haplotype, known as 8c mice, while murine tau is knocked out by a targeted disruption of exon 1 (Duff et al., 2000; Andorfer et al., 2005). Mice were bred on a C57BL/6 background. Breeding pairs were provided by the Research Foundation for Mental Hygiene (RFMH) via Jackson Laboratories [B6.Cg-Mapt^tm1(EGFP)Klt^ Tg(MAPT)8cPdav/J; JAX #005491]. Hemizygous animals of mixed sex and their non-transgenic (ntg) littermates that expressed neither htau nor murine tau were used. Mice were bred and housed under identical conditions in individually ventilated cages on standard rodent bedding (Rettenmayer^®^, Germany) in the AAALAC-accredited animal facility of QPS Austria GmbH. Cotton nestlets (Plexx^®^) were provided as nesting material. The room temperature was kept at approximately 21°C and the relative humidity between 40–70%. Mice were housed in same sex groups of up to four animals under constant light-cycle (12 h light/dark). If animals had to be separated due to fighting, the single housed animal received wood wool as additional nesting material. Dried pelleted standard rodent chow (Altromin^®^, Germany) and normal tap water were provided *ad libitum*. Each individual animal was checked regularly for any clinical sign. During weaning animals were ear punched for identification and the removed tissue used for genotyping. Male and female animals of equal number were used. Actual animal numbers are given in the figure legends.

Animal studies conformed to the Austrian guidelines for the care and use of laboratory animals (Tierversuchsgesetz 2012-TVG 2012, BGBl. I Nr. 114/2012). Animal housing and euthanasia were approved by the Styrian government (Amt der Steiermärkischen Landesregierung, Abteilung 13 – Umwelt und Raumordnung Austria; ABT13-78Jo115/2013–2016; ABT13-78Jo-118/2013-13).

### Tissue Sampling and Preparation

All mice were anesthetized by intraperitoneal injection of 600 mg/kg pentobarbital. Once animals were deeply anesthetized, the thorax was opened and blood was drawn by heart puncture of the left ventricle and collected in lithium heparin tubes. Mice were afterward transcardially perfused with physiological (0.9%) saline. The left hemisphere of each animal was immediately frozen on dry ice and stored at −80°C until further analysis. The right hemisphere was immediately fixed in freshly prepared 4% paraformaldehyde in 0.1 M phosphate buffer (pH 7.4) for 2 hours (h) at room temperature (RT), subsequently cryo-protected in 15% sucrose/phosphate buffered saline (PBS) solution over night at 4°C and then snap-frozen in dry ice-cooled isopentane. Sagittal brain sections were systematically cut at 10 μm thickness using a cryostat (CM 3050 S, Leica). For each immunofluorescence labeling a uniform systematic random set of five sections per mouse was used to analyze ptau throughout the hemisphere.

### Immunofluorescence Labeling for ptau

Cryosections were air-dried for 45 min, washed in Dulbecco’s PBS (DPBS, Pan Biotech, PO4-360000) and then treated with 1x citrate buffer (AP-9003, Thermo Scientific) for 15 min at 95°C. After cooling to RT murine sections were blocked in a M.O.M. Blocking Reagent (BMK-2202, Vector Laboratories) in 0.3% Triton X-100/PBS for 1 h at RT. Afterward, tissue sections were incubated with primary antibodies (rabbit monoclonal anti-pTau Ser202 [EPR2402], 1:1500, Abcam, ab108387, stable public ID# AB_10860874; mouse monoclonal anti-pTau Ser396 [PHF13], 1:1000, Cell Signaling Technology, #9632, stable public ID# AB_2266237) over night at 4°C. Primary antibody binding was visualized by labeling with the corresponding fluorophore-conjugated secondary antibodies (DyLight 650-conjugated donkey anti-rabbit IgG (H+L), 1:500, abcam, ab96922 to label EPR2402; Alexa Fluor 647-conjugated donkey anti-mouse IgG (H+L), 1:500, Abcam, ab150111 to label PHF13) for 1 h at RT. Cell nuclei were then counterstained using a 4′,6-diamidin-2-phenylindol solution (DAPI, AppliChem, A1001) and sections were washed in PBS and distilled water before covering with Mowiol (Sigma Aldrich, #81381) and coverslips.

Between experimental steps, tissue sections were washed with DPBS for 5 min at least twice. All steps during and after the use of fluorophore-conjugated antibodies were performed in the dark.

### Imaging

Mosaic images of the immunolabelled sections were recorded on a Zeiss automatic microscope AxioScan Z1 with high aperture lenses, equipped with a Zeiss Axiocam 506 mono and a Hitachi 3CCD HV-F202SCL camera and Zeiss ZEN 2.3 software. Multichannel images were converted to full resolution gray-scale single channel tif files that were subsequently used for quantitative image analysis with Image Pro Plus (v6.2) software. The cortex and hippocampus were identified by drawing areas of interest (AOI) on the images. The background was corrected by subtraction of the lowpass-filtered image, and immunoreactive objects were then detected by adequate thresholding and morphological filtering. Different object features were then quantified, among them the percentage of cumulative object area based on AOI size (immunoreactive area; this is the most comprehensive parameter indicating whether there are differences in overall immunoreactivity), and the number of objects normalized to AOI size (numerical object density).

### Biochemistry

Tissue was homogenized in 9 volumes (9 μl per mg) of cold extraction buffer 1 (25 mM Tris–HCL pH = 7.4, 150 mM NaCl, 1 mM EDTA, 1 mM EGTA, 10 mM ß-glycerophosphate, 30 mM NaF, 2 mM Na3VO4, protease and phosphatase inhibitor cocktails) and centrifuged at 80,000 g for 15 min at 4°C. Aliquots of the resulting supernatants were collected as soluble fraction and frozen at −80°C. To prepare sarcosyl-insoluble fraction, the pellets were resuspended in extraction buffer 2 (10 mM Tris–HCl pH = 7.4, 800 mM NaCl, 300 mM sucrose, 1 mM EGTA, protease and phosphatase inhibitor cocktails; same volume as above) and centrifuged at 4,000 × *g* for 10 min at 4°C. Supernatants were transferred to fresh tubes and sarcosyl (30% aqueous solution) was added to a final concentration of 1% and incubated for 1.5 h at RT. After centrifugation at 80,000 × *g* for 30 min at 4°C, supernatants were discarded and the pellets resuspended in buffer 3 as insoluble fraction (50 mM Tris–HCl, pH = 7.4; same volume as above).

Samples were afterward analyzed with the Duplex Tau (pT231)/Total Tau kit and the pTau Thr181 kit (Mesoscale Discovery, Gaithersburg, MD, United States) as recommended by the manufacturer. Due to the lack of stable ptau calibrators the signals are given as arbitrary units (AU).

### Statistics

Data analysis was performed in GraphPad Prism^TM^ 8.1 (GraphPad Software Inc., United States). Dot plot graphs show individual and groups means, and standard error of the mean (SEM). The significance level was set at *p* < 0.05. Group means were compared using One-way or Two-way analysis of variance (ANOVA) with a subsequent *post hoc* test after evaluation for Gaussian distribution by Kolmogorov-Smirnov test. The utilized statistical tests and exact sample numbers are mentioned in the figure legends.

### Raw Data

All raw data collected and evaluated for this manuscript are listed in Supplementary Table 1.

## Results

**FIGURE 1:**
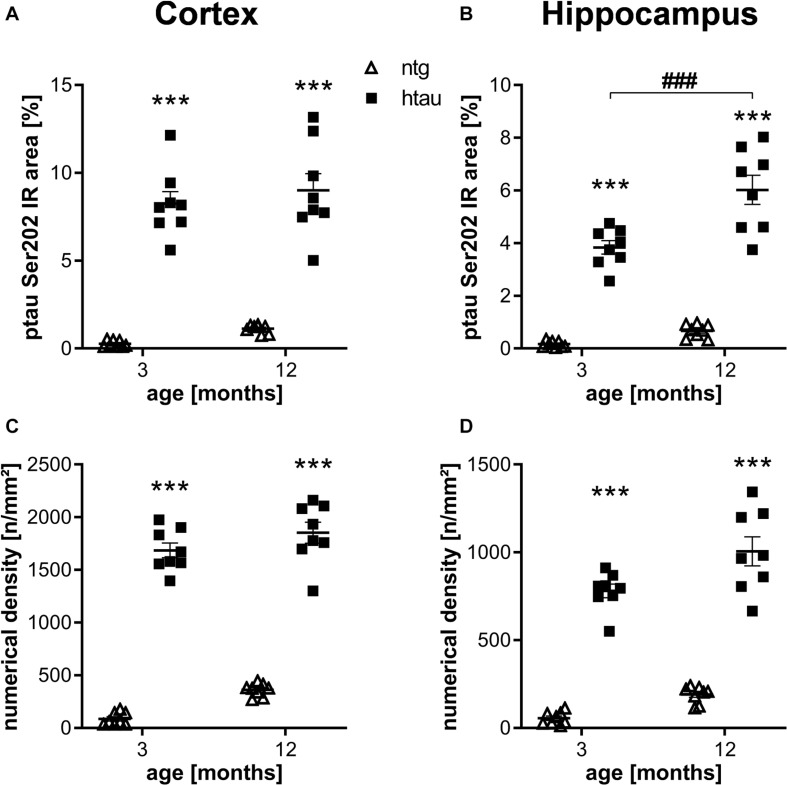
Quantification of ptau Ser202 levels in the cortex and hippocampus of 3 and 12 month old htau mice compared to non-transgenic (ntg) littermates by EPR2402 antibody. **(A,B)**: Immunoreactive area in percent in the cortex **(A)** and hippocampus **(B)**. **(C,D)**: Object density in n/mm2 in the cortex **(C)** and hippocampus **(D)**. *n* = 8 per group, 5 slides per animal. Mean + SEM; Two-way ANOVA with Bonferroni’s *post hoc* test; ***p < 0.001. *differences between genotypes; ^#^differences between age groups.

For quantification of ptau at different residues in htau mice we analyzed animals of different age and sex using histological and biochemical methods. Ntg animals were labeled in parallel to determine the background signal from autofluorescence and potential unspecific binding of primary and secondary antibodies. Sex differences could not be observed; groups were therefore merged to increase group sizes. In a first step, ptau Ser202 was quantified in 3 and 12 month old htau mice using the EPR2402 antibody. Quantification of the immunoreactive (IR) area in the cortex and hippocampus showed strong ptau Ser202 signal in htau mice, whereas only weak general background was detected in ntg littermates (Figures 1A,B), suggesting that the antibody specifically binds ptau Ser 202. While the signal in the cortex was already strong at 3 month of age, the signal in the hippocampus did significantly increase from 3 to 12 months. Additional analysis of the numerical density revealed similar results than the IR area, the ptau Ser202 numerical density in the cortex and hippocampus of htau mice was already significantly increased at 3 month of age (Figures 1C,D). Differences between age groups could not be detected.

**FIGURE 2:**
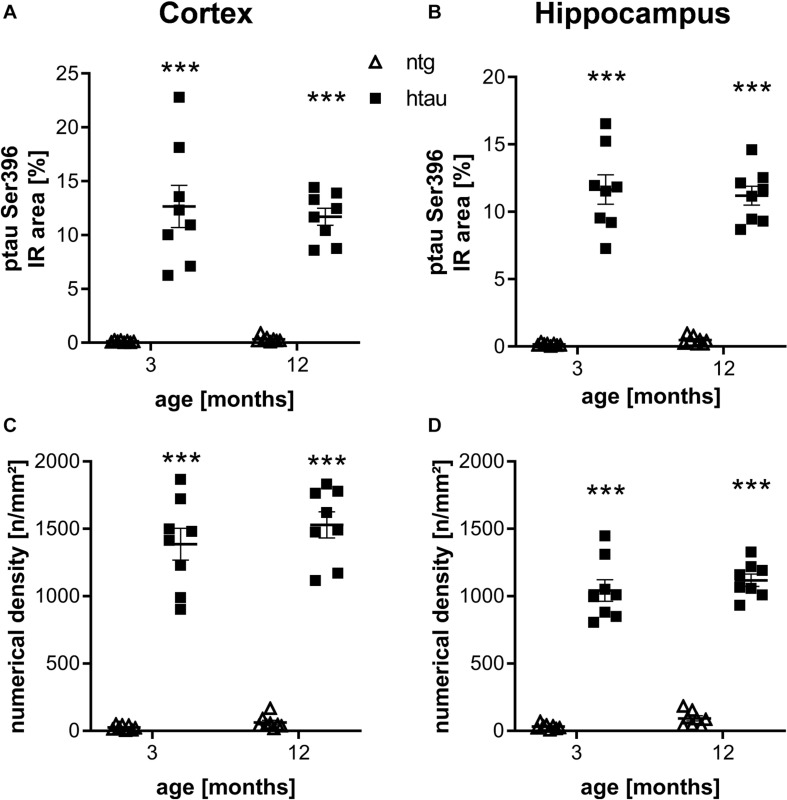
Quantification of ptau Ser396 levels in the cortex and hippocampus of 3 and 12 month old htau mice compared to ntg littermates by PHF13 antibody. **(A,B)**: Immunoreactive area in percent in the cortex **(A)** and hippocampus **(B)**. **(C,D)**: Object density in n/mm^2^ in the cortex **(C)** and hippocampus **(D)**. *n* = 8 per group, 5 slides per animal. Mean + SEM; Two-way ANOVA with Bonferroni’s *post hoc* test; ***p < 0.001.

Further histological analysis of htau mice using the ptau Ser396 specific PHF13 antibody resulted also in a large percentage of IR area and high numerical density of immunoreactive objects in the cortex and hippocampus of 3 and 6 month old htau mice compared to ntg littermates (Figures 2A–D), again suggesting high specificity of the primary and secondary antibodies. No changes over time could be observed.

**FIGURE 3:**
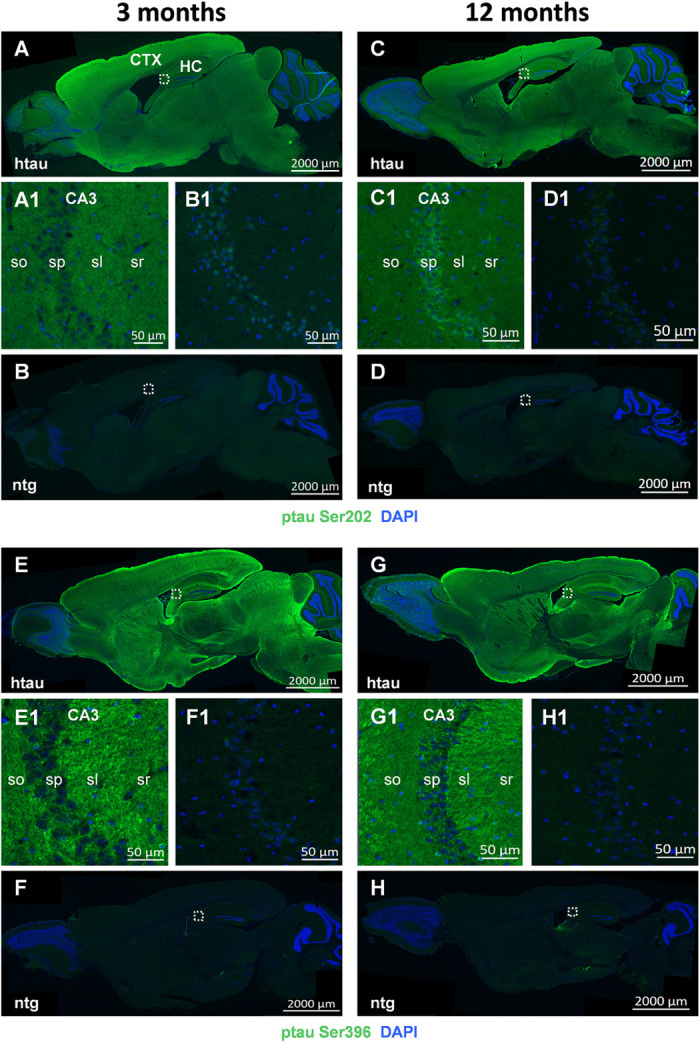
Representative images of ptau Ser202 and ptau Ser396 in the brain of 3 and 12 month old htau mice. Immunofluorescent labeling of ptau Ser202 (**A–D**; green) and ptau Ser396 (**E–H**; green) on sagittal sections of 3 **(A,E)** and 12 **(C,G)** month old htau mice compared to age-matched ntg controls **(B,F,D,H)**. Very high signal is evident in the frontal cortex of htau mice. Immunofluorescence is absent from ntg tissue except for minor unspecific signal in the nuclei. Magnified areas in **(A1–H1)** show hippocampal CA3 region as indicated in the whole slide scan. Nuclei are labeled by DAPI (blue). So, stratum oriens; sp, stratum pyramidale; sl, stratum lucidum; sr, stratum radiatum. Scale bar: **(A–H)**: 2,000 μm. **(A1–H1)**: 50 μm.

Representative images of ptau Ser202 and ptau Ser396 in 3 and 12 month old animals are shown in Figures 3A–H, respectively. Images of entire sagittal brain sections of htau mice show that the regional distribution of ptau Ser202 differs compared to ptau Ser396. In 3 month old htau mice, ptau Ser202 signal is very strong all over the cortex but lower in the midbrain and brainstem (Figure 3A). Ptau Ser396 signal is at the same time strong in distinct cortical, hippocampal and striatal regions as well as in discrete midbrain and brainstem areas (Figure 3E). The signal of both ptau sites does not change compared to 12 month old animals (Figures 3C,G, respectively). Signal of both ptau residues is barely visible in the olfactory bulb and cerebellum (Figures 3A,C,E,G) as well as in ntg animals (Figures 3B,D,F,H). Negative controls of ptau Ser202 and ptau Ser396 labellings are shown in Supplementary Figure S1.

**FIGURE 4:**
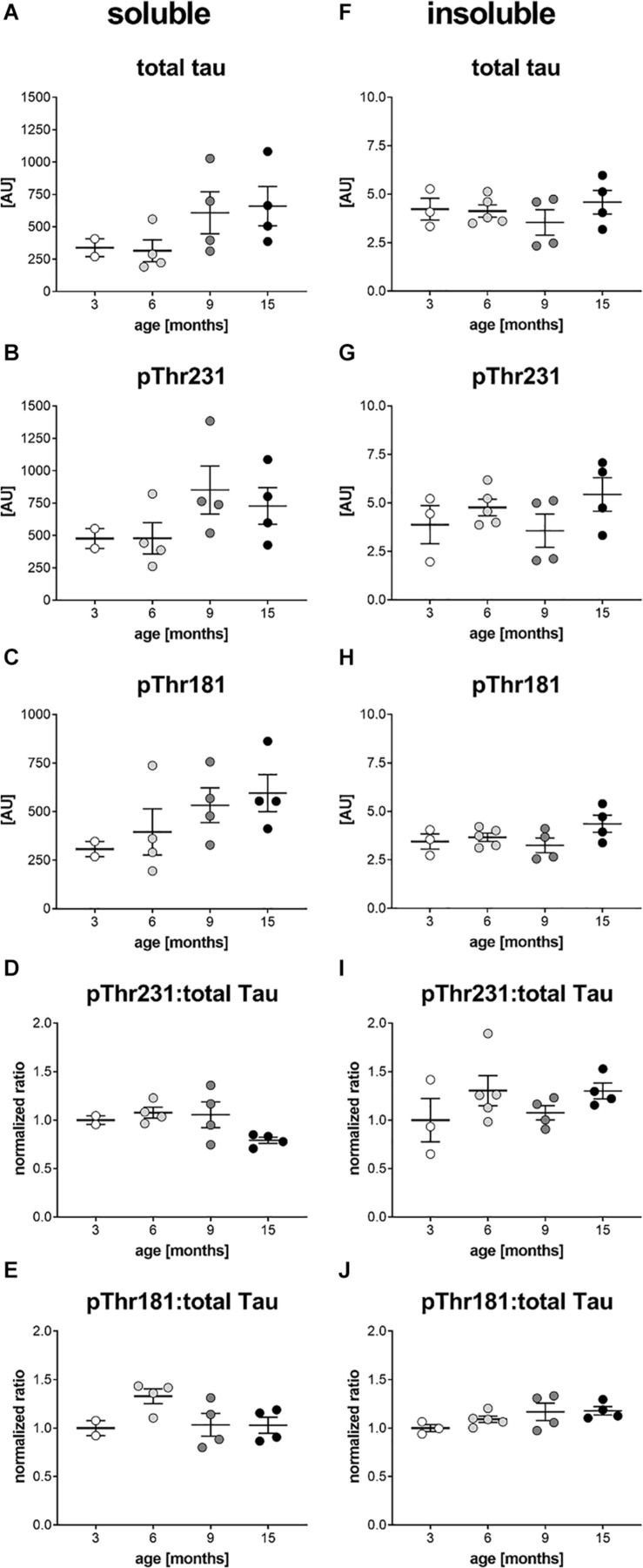
Soluble and insoluble total and phosphorylated tau levels in the brain of htau mice. Soluble **(A–C)** and insoluble **(F–H)** tissue fractions of the left hemisphere of 3, 6, 9, and 15 month old htau mice were analyzed for total tau **(A,F)**, ptau Thr231 **(B,G)**, and ptau Thr181 **(C,H)** levels by Mesoscale Discovery immunosorbent assay. Ratio of soluble and insoluble total and ptau Thr231 is shown in (**D** and **I**), respectively. Ratio of soluble and insoluble total and ptau Thr181 is shown in (**E** and **J**), respectively. **(D,E,I,J)**: Ratios were calculated and values normalized to mean of 3 month group. One-Way ANOVA with Newman Keul’s multiple comparison test. Mean ± SEM; *n* = 2–5.

In a next step, brain homogenates of 3, 6, 9, and 15 month old htau mice were processed into soluble and insoluble fraction and analyzed for total tau, ptau Thr231 and ptau Thr181 levels by immunosorbent assay. Since in ntg mice, that neither express the human tau nor the murine tau, the ptau signal was below detection limit (data not shown) ntg animals were omitted from the following analyses. Analysis of total tau, ptau Thr231 and ptau Thr181 in the soluble fraction showed the same trend over age. Total tau (>200 AU) and phosphorylation at both residues (>200 AU) was already strong at the age of 3 months (Figures 4A–C). Presenting the ratio of ptau Thr231 and total tau as well as ptau Thr181 and total tau showed that ptau levels did not increase over age (Figures 4D,E). Differences between age groups were not significant as analyzed by One-way ANOVA. Analysis of total tau, ptau Thr231 and ptau Thr181 in the insoluble fraction showed no differences between age groups, except for a slight, but not significant increase of both ptau residues over age when presented as ratio of ptau over total tau (Figures 4F–J). Absolute insoluble total (∼ 5 AU) and ptau values (∼ or < 5 AU) were much lower compared to the soluble fraction (>200 AU; Figure 4).

## Discussion

Our analysis of the tau phosphorylation profile of htau mice showed strong levels of tau phosphorylation at residue Ser202 using the EPR2402 antibody already at the age of 3 months. The original study introducing htau mice used the CP13 antibody to evaluate ptau pSer202 levels showing phosphorylation qualitatively in 16 month old animals (Andorfer et al., 2005; Flunkert et al., 2013). The results of this study were validated using the same antibody in 11 month old htau mice (Acker et al., 2013). Since animals analyzed in these studies have probably a different background and different antibodies were used, a direct comparison is difficult. Geiszler and colleagues quantified ptau Ser202 levels in htau mice on a C57Bl/6J background by using the CP13 antibody in Western blots. Their results suggest that ptau Ser202 is already highly phosphorylated in 2 month old htau mice and thus validating our histological results (Geiszler et al., 2016).

Evaluation of ptau Ser396 levels in htau mice by PHF13 antibody also revealed strong levels already at 3 month of age. Previous studies used always the PHF1 antibody that labels ptau only when phosphorylated at ptau Ser396 and ptau Ser404 at the same time. The antibody is thus not as useful as the PHF13 antibody for studying phosphorylation of ptau Ser396 only (Andorfer et al., 2005; Acker et al., 2013; Forest et al., 2013; Sankaranarayanan et al., 2015; Geiszler et al., 2016). Although specificity differs between antibodies, observed ptau Ser396 levels in young animals are comparable between studies (Geiszler et al., 2016).

To evaluate the translatability of tau phosphorylation in htau mice compared to human tauopathies, we further evaluated levels of ptau Thr231 and Thr181 in the soluble and insoluble brain fraction of 3 to 15 month old htau mice by immunosorbent assay. Our results show that ptau Thr231 levels are phosphorylated in the soluble brain fraction already at the age of 3 months, suggesting that phosphorylation starts even earlier and thus reflecting the human disease (Luna-Munoz et al., 2005). Tau phosphorylation at residue Thr181 was recently shown to be a valuable non-invasive diagnostic biomarker since changes can not only be measured in patients’ CSF but also the plasma. In htau mice we observed similar ptau Thr181 levels in the soluble brain fraction compared to ptau Thr231 levels, suggesting an early increase similar to the human disease (Janelidze et al., 2020).

Analysis of the insoluble brain fraction for total and ptau revealed no changes over age. These results are in agreement with results of the original publication about htau mice, showing that animals do not develop NFTs and thus insoluble tau (Andorfer et al., 2005). For the analysis of the soluble and insoluble brain fraction we were able to analyze only a small number of animals. Although a trend toward an increase of ptau Thr181 over age could be observed in the soluble brain fraction, changes were not significant. To strengthen the value of these results, evaluation of a larger number of animals is recommended.

Since ptau Thr231, Thr181, Ser202, and Ser396 levels are shown here to be strong in the cortex and hippocampus already at the age of 3 months by histological analyses as well as immunosorbent assay and the latter in the cortex at the age of 2 months by Western blotting (Geiszler et al., 2016), it would be of great interest to analyze even younger animals to evaluate the age of pathology onset and progression. So far, htau mice were already frequently used for efficacy studies of new compounds against tauopathies. Almost all of these studies started treatment in young htau mice and evaluated the effect in old animals or even used old mice from the beginning on (Ma et al., 2013; Congdon et al., 2016; Maphis et al., 2016; Giannopoulos et al., 2018; Brendel et al., 2019). Only in two studies treatment of htau mice started before the age of 4 months and evaluated compound effects before the age of 6.5 months (Garwood et al., 2010; Davidowitz et al., 2020). Garwood and colleagues were able to reduce cortical pro-inflammatory cytokines in htau mice by treating 3–4 month old htau mice for only 14 days with minocycline and thus showing that the pathology of htau mice can be improved by early treatment. According to the here presented data, ptau pathology starts even before the age of 3 months, so to observe preventive treatment effects the use of very young animals at study start would be recommended.

## Conclusion

Our results show that tau phosphorylation at residues Ser202, Ser396, Thr231, and Thr181 is an early event in the brain of htau mice on a C57Bl/6J background. Htau mice on a C57Bl/6J background may thus be used already at young age for efficacy studies to analyze new compounds against tauopathies.

## Data Availability Statement

All datasets presented in this study are included in the article/Supplementary Material.

## Ethics Statement

The animal study was reviewed and approved by Amt der Steiermärkischen Landesregierung, Abteilung 13 – Umwelt und Raumordnung Austria.

## Author Contributions

JN designed, planned, performed and analyzed histological experiments, and edited the manuscript. MD prepared figures and wrote and edited the manuscript. TL designed, performed and analyzed all biochemical experiments, and edited the manuscript. SA analyzed histological experiments and prepared figures. SF prepared figures, interpreted results, and wrote the manuscript. BH-P conceived the study, interpreted results, and edited the manuscript. All authors contributed to the article and approved the submitted version.

## Conflict of Interest

All authors are employees of QPS Austria GmbH.

## Abbreviations

AAALAC: Association for Assessment and Accreditation of Laboratory Animal Care
AD: Alzheimer’s Disease
ANOVA: Analysis of variance
APP: Amyloid Precursor Protein
CNS: central nervous system
CSF: cerebrospinal fluid
ntg: non-transgenic
MAPT: microtubule associated protein tau
PAC: P1-derived artificial chromosome
RFMH: Research Foundation for Mental Hygiene
SEM: Standard error mean
TUNEL: TdT-mediated dUTP-biotin nick end labeling.
