# Supplementary material for: Constant Levels of Tau Phosphorylation in the Brain of htau Mice
Source: Front Mol Neurosci. 2020 Aug 28;13:136. doi: 10.3389/fnmol.2020.00136 (PMC7485327; doi:10.3389/fnmol.2020.00136)
Supplement: Supplementary file 1 [file Image_1.pdf]

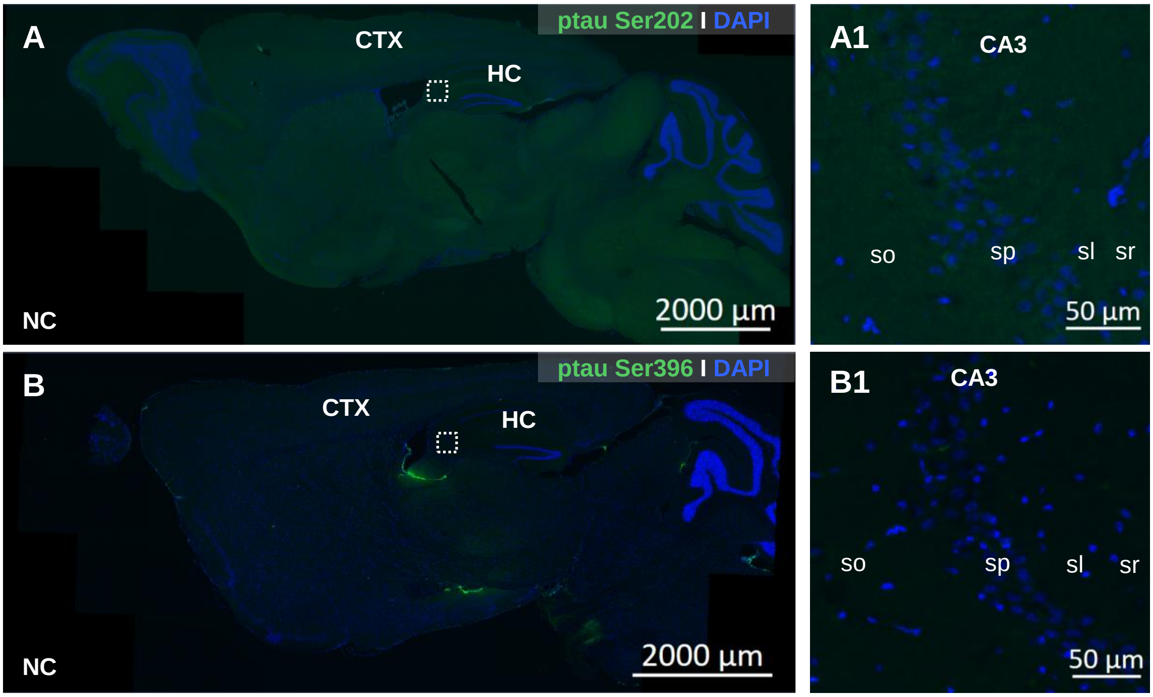

Supplementary Figure 1: Representative images of negative controls for the immunofluorescent labeling of ptau Ser202 and ptau Ser396 in the brain of 3 month old htau mice as shown in Figure 3. Immunofluorescent labeling of sagittal sections of 3 month old htau mice omitting the primary ptau Ser202 (**A** green) and ptau Ser396 (**B**; green) antibody. Minor unspecific green signal can be observed in the negative control for ptau Ser202 labeling (**A, A1**). Magnified areas in A1 and B1 show hippocampal CA3 region as indicated in the whole slide scan. Nuclei are labeled by DAPI (blue). CTX: cortex; HC: hippocampus; so: stratum oriens; sp: stratum pyramidale; sl: stratum lucidum; sr: stratum radiatum. Scale bar: **A, B**: 2.000 µm. **A1, B1**: 50 µm.
